# Determinants of the Usage of Splice-Associated *cis*-Motifs Predict the Distribution of Human Pathogenic SNPs

**DOI:** 10.1093/molbev/msv251

**Published:** 2015-11-05

**Authors:** XianMing Wu, Laurence D. Hurst

**Affiliations:** ^1^Milner Centre for Evolution, Department of Biology and Biochemistry, University of Bath, Bath, Somerset, United Kingdom

**Keywords:** pathogenic SNPs, splicing *cis*-motif, splice site, exonic splicing enhancer

## Abstract

Where in genes do pathogenic mutations tend to occur and does this provide clues as to the possible underlying mechanisms by which single nucleotide polymorphisms (SNPs) cause disease? As splice-disrupting mutations tend to occur predominantly at exon ends, known also to be hot spots of *cis*-exonic splice control elements, we examine the relationship between the relative density of such exonic *cis*-motifs and pathogenic SNPs. In particular, we focus on the intragene distribution of exonic splicing enhancers (ESE) and the covariance between them and disease-associated SNPs. In addition to showing that disease-causing genes tend to be genes with a high intron density, consistent with missplicing, five factors established as trends in ESE usage, are considered: relative position in exons, relative position in genes, flanking intron size, splice sites usage, and phase. We find that more than 76% of pathogenic SNPs are within 3–69 bp of exon ends where ESEs generally reside, this being 13% more than expected. Overall from enrichment of pathogenic SNPs at exon ends, we estimate that approximately 20–45% of SNPs affect splicing. Importantly, we find that within genes pathogenic SNPs tend to occur in splicing-relevant regions with low ESE density: they are found to occur preferentially in the terminal half of genes, in exons flanked by short introns and at the ends of phase (0,0) exons with 3′ non-“AGgt” splice site. We suggest the concept of the “fragile” exon, one home to pathogenic SNPs owing to its vulnerability to splice disruption owing to low ESE density.

## Introduction

Although it is clear that many disease-causing mutations are nonsense or nonsynonymous changes within exons, that many synonymous mutations also cause disease (Chamary et al. 2006; Sauna and Kimchi-Sarfaty 2011; Hunt et al. 2014; Bali and Bebok 2015) suggests that mechanisms beyond replacement of one amino acid for another (or for a stop), can be important. That synonymous mutations cause disease also suggests that many nonsynonymous mutations might have their effects for reasons other a slightly altered amino acid content of proteins.

One of the predominant mechanisms by which synonymous mutations cause disease (and affect fitness more generally) is via modulation of splicing (Faustino and Cooper 2003; Chamary et al. 2006). Indeed, it is estimated that of known disease-associated synonymous variants perhaps over 90% impact splicing (Mort et al. 2014). In some cases, the effect is a simple disruption of the splice site. It is for example estimated that 15% of splice site mutations could lead to human genetic disease (Krawczak et al. 1992). However, splice sites, while important, do not contain all the information for splicing in humans (Wang and Burge 2008). Indeed, in the human genome approximately 50% of the information defining splice sites is in *cis*-motifs, typically in close proximity to the splice sites (Lim and Burge 2001). More generally, the behavior of these *cis*-motifs is dependent on the intragene location, with one motif having different activity dependent on local context and position within the exon (Wang and Burge 2008; Ke et al. 2011). Detailed studies suggest that for some exons, 30% of individual mutations in a given exon can affect the splice pattern (Pagani et al. 2005). In turn, it is expected that disruption of *cis*-motifs might also cause disease, a prediction borne out by the evidence (Nissim-Rafinia and Kerem 2002; Faustino and Cooper 2003). For instance, a splicing enhancer disruption in exon 51 of *FBN1* gene relates to Marfan syndrome (Caputi et al. 2002) and a point mutation in exon 7 of *SMN2* gene is associated with Motor Neurone Disease (Gavrilov et al. 1998; Cartegni and Krainer 2002; Wirth et al. 2006).

The possible selective importance of *cis*-motifs is underscored by the observed selection to preserve them at the terminal regions of exons. Many studies have reported that selective constraints are more common at the ends of exons due to the splicing control, such that new mutations at exon ends are likely to be eliminated by purifying selection, even in species with low effective population size (Majewski and Ott 2002; Fairbrother et al. 2004; Carlini and Genut 2006; Parmley et al. 2006, 2007; Cáceres and Hurst 2013; Wu and Hurst 2015). To date, two important exonic splicing control elements, serving as enhancers (exonic splicing enhancers, ESEs) (Blencowe 2000) and silencers (exonic splicing silencers, ESSs) (Amendt et al. 1995; Kan and Green 1999), have been investigated. These *cis*-motifs generally reside within exon ends and function by interacting with certain regulators (SR proteins and hnRNP) (Zheng et al. 2000; Rowen et al. 2002). With little evidence that ESS motifs are under purifying selection (Chamary et al. 2006; Parmley and Hurst 2007; Parmley et al. 2007), while ESEs generally are, we here concentrate our attention on ESEs. It is estimated that about 4–5% of synonymous mutations are under selection in humans because they disrupt ESEs (Cáceres and Hurst 2013). In particular, we ask whether the biases in intragene distribution of ESEs predicts in any manner biases in the intragene distribution of pathogenic single nucleotide polymorphisms (SNPs). Such a covariance would lend support to the postulate that splice disruption is an important mechanism for pathogenesis (Cartegni et al. 2002). It would also suggest that we ignore synonymous SNPs for diagnostics at our peril.

The notion that ESE location might predict disease causing SNP distributions has some support. Recently, splice-affecting SNPs (likely to be enriched for pathogenic mutations) were found to be significantly enriched at exon ends (Woolfe et al. 2010), the hot spots of ESEs (Nelson and Green 1988; Lavigueur et al. 1993; Graveley et al. 1998; Fairbrother et al. 2004; Carlini and Genut 2006; Parmley et al. 2006, 2007; Cáceres and Hurst 2013). Therefore, for understanding patterns of molecular evolution, for understanding splice control and for understanding how and why mutations cause disease, it is helpful to have a robust understanding of the relationship between ESE usage and distribution of disease-causing mutations. Here, we address this by observing the coupling of ESE density with relative abundance of exonic pathogenic SNPs.

ESE usage within and between genes is known to vary with numerous factors. As noted above, ESEs tend to be at their most dense in the terminal (up to 70–100 bp) portion of exons (Fairbrother et al. 2004; Cáceres and Hurst 2013). Moreover, ESE usage has recently been shown to be higher in the 5′-exons compared with 3′-exons (Wu and Hurst 2015) and in exons flanking large introns (Dewey et al. 2006; Cáceres and Hurst 2013; Wu and Hurst 2015), as well as in genes with high exon density (number of exons per coding bp) (Wu and Hurst 2015). These covariates were conjectured to reflect selection to minimize the impact of possible decoy splice sites (Wu and Hurst 2015). This selection, it was conjectured, would be more common for earlier exons (more downstream splice sites), exons flanked by larger introns (more possibilities for decoys) and in genes with more exons (more splice sites, hence more decoys for any given splice site; Wu and Hurst 2015).

ESE usage is also a function of the flanking splice site (Berget 1995; Graveley 2000), a higher ESE density being considered necessary to support the reinforcement of the flanking “weak” splice sites (Fairbrother et al. 2002; Dewey et al. 2006; Plass et al. 2008; Cáceres and Hurst 2013). This too can fit into the broader decoy splice site model, in the sense that if the weak splice site is not found a decoy (inappropriate) one might be. Recently, we found usage of splicing *cis*-motifs correlates well and positively with the usage of tetra-nucleotide splice sites “AGgt” and “agGT” (nucleotides of exons in upper case, nucleotides of introns in lower case) across 30 species (Wu and Hurst 2015).

In further examination we noted, serendipitously, that exon phase appears to be a predictor of ESE density (for evidence see below). Phase here refers to where in the codon the splice site hits, a phase zero exon end being one cut between whole codons. The three possible phases are not found in equal proportions (Fedorov et al. 1992; Long et al. 1995; Ruvinsky et al. 2005). Furthermore, in phylogenetically manner, we observe a significant and negative correlation between proportion of phase zero splice site and usage of ESEs (table 1). To date, we have no good explanation as to why phase might matter, but for the purposes of this article such considerations are not relevant. We simply consider it to be a correlate to ESE density.

**Table 1.** *Cis*-Motif Usage Correlates Significantly with Proportion of Phase Zero Splice Sites.

	All Exons (AA)	All Exons (codon)	Random 5,000 Exons (AA)	Random 5,000 exons (codon)
**Log BF****^a^**** (Y∼P_phase-zero_)**	140.6781	103.9811	82.6049	114.0758
**R Trait 1 2****^b^**	< 0	<0	<0	<0

Note.—Y, proportion of amino acids/codons showing significant trends; P_phase-zero_, proportion of intercodon splice site.

^a^Log BF (log Bayes factor) = 2*(log [harmonic mean (complex model)]−log [harmonic mean (simple model)]), All Log BF values in the table are >10, so the evidences of all correlations are very strong.

^b^BayesTraits parameter “R Trait 1 2” can indicate whether the correlation is positive (>0) or negative (<0).

Given the above we hence consider the intragenic location of pathogenic SNPs and five possible covariates: 1) relative position in exons, 2) relative position in genes, 3) flanking intron size, 4) usage of splice sites, and 5) exon end phases. For each we consider whether pathogenic SNPs are more or less likely to occur where ESEs are more or less common. As many genes are not associated with diseases (either because they are too essential or too unimportant), we consider the trends within the class of genes within which disease-associated SNPs are found. We start, however, by asking whether disease causing genes are at all unusual in the exon–intron architecture.

## Results

### Disease-Associated Genes Tend to Have More Exons

Before considering intragenic trends we first ask whether genes that contain pathogenic SNPs are intrinsically those most likely to be affected by splice-related mechanisms. Were pathogenic SNPs to have their effect via missplicing, we might expect that genes with more exons (more splice sites) tend to be disease-associated. According to disease-related information of sequence variation in Clinvar database (http://www.ncbi.nlm.nih.gov/clinvar/, last accessed November 24, 2014; Landrum et al. 2014), we established a data set of 9,818 pathogenic SNPs coming from missense and silent mutations (not nonsense) (supplementary table S1, Supplementary Material online). Of these SNPs 8,250 (84%) exist in internal exons. From UCSC (Karolchik et al. 2004) (http://genome.ucsc.edu/cgi-bin/hgTables, last accessed November 24, 2014), 1,747 gene sequences that contain these pathogenic SNPs were derived. Note that here and elsewhere we exclude nonsense SNPs, as their likely mode of pathology is probably not splice related (but see below).

Consistent with the splice decoy model for determining the genic richness of splicing-related *cis*-motifs, we found that exon number correlates significantly with ESE usage (Wu and Hurst 2015). In turn, we find that the absolute number of exons in disease genes is higher than that in nondisease gene (Mann–Whitney *U* test: *P* = 5.87 × 10^−^^119^; median of number of exons in disease genes = 12, median of number of exons in nondisease genes = 8). However, under the null that all bases in all coding sequences (CDSs) have the same likelihood of causing disease, we expect long genes to be more likely to be disease associated (just because they have more base pairs). This is indeed the case (Mann–Whitney *U* test: *P* = 2.41 × 10^−^^103^; median of CDS size in disease genes = 1,689 bp, median of CDS size in nondisease genes = 1,248 bp). So here we ask whether disease-associated genes have more exons when CDS length is controlled.

As the relationship between CDS length and number of exons is not linear, we employ a Mann–Whitney *U* test to analyze residuals of a loess regression for number of exons in all genes (disease and nondisease genes) against CDS length. We find that the number of exons in disease-associated genes, controlling for CDS size by this method, is higher than that of nondisease genes (Mann–Whitney *U* test: *P* = 8.11 × 10^−^^23^, median of residuals for disease genes: 0.921, median of residuals for nondisease genes: 0.146) (supplementary table S2, Supplementary Material online). One interpretation of this is that the more exons in a gene the more likely an inappropriate splice event might take place. However, as exon size and expression level covary, this interpretation is by no means unique.

### Both Splicing *cis*-Motifs and Pathogenic SNPs Tend to Be Present at Ends of Exons

We now turn to intragenic predictors of where non-nonsense pathogenic SNPs are over or underrepresented. It has been found that *cis*-exonic splice control elements, such as ESEs, tend to enriched at the ends of exons (Nelson and Green 1988; Lavigueur et al. 1993; Graveley et al. 1998; Fairbrother et al. 2004; Carlini and Genut 2006; Parmley et al. 2006, 2007; Cáceres and Hurst 2013). This is at least part of the explanation for reduced substitution rates and rarity of SNPs at the ends of exons (Majewski and Ott 2002; Fairbrother et al. 2004; Carlini and Genut 2006; Parmley et al. 2006, 2007; Cáceres and Hurst 2013; Wu and Hurst 2015). Consistent with this, splice-affecting variants (many of which we expect to be pathogenic), are significantly enriched at both ends of exons (Woolfe et al. 2010). We thus ask whether pathogenic SNPs in internal exons (not first and last exons) are more common at exon ends.

**Fig. 1.**
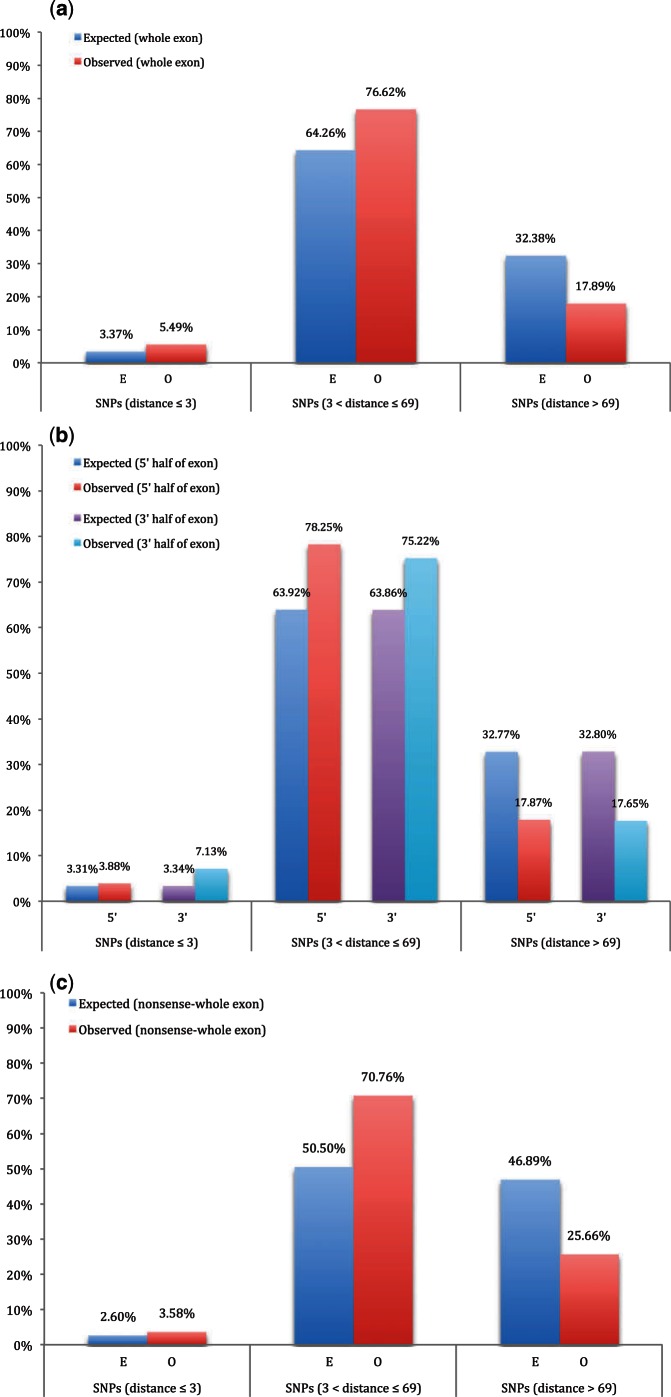
Pathogenic SNPs are enriched close to exon junctions. (*a*) Of 8,250 pathogenic SNPs in internal exons, the great majority (76.62%) are within 3–69 bp from the exon ends. We consider enrichment of SNPs in three domains: 1) splice sites (≤3 bp) are greatly enriched for pathogenic SNPs (Observed: 5.49%, Expected: 3.37%); 2) Pathogenic SNPs have significant preference at exon terminal domains (3–69 bp, Observed: 76.62%, Expected: 64.26%). 3) Distribution of pathogenic SNPs in exon cores are relatively underrepresented (Observed: 17.89%, Expected: 32.38%). (χ^2 ^= 841.64, df = 2, *P* < 1.74 × 10^−183^). (*b*) The same pattern of significant deviation in pathogenic SNPs distribution are observed for: 1) 5′-half of internal exons (χ^2 ^= 410.62, df = 2, *P* < 6.85 × 10^−90^); 2) 3**′**-half of internal exons (χ^2 ^= 551.74, df = 2, *P* < 1.55 × 10^−120^). (*c*) Distribution of nonsense pathogenic SNPs in internal exons are similar to that of non-nonsense mutations (χ^2 ^= 455.37, df = 2, *P* < 1.31 × 10^−99^).

Of the 8,250 pathogenic SNPs in internal exons, the great majority (76.62%) are within 3–69 bp from the ends of the internal exons. This statistic, however, is uninformative without assessment of relative enrichment. To this end, we consider enrichment of SNPs in three domains: within 3 bp of splice sites; between 3 and 69 nt from exon ends; all other sequence from internal exons, that is, exon core. Comparing the distribution of pathogenic SNPs in the exons against the expected distribution in the same SNP bearing exons, we find that splice sites (≤3 bp) are greatly enriched for pathogenic SNPs (Observed: 5.49%, Expected: 3.37%), as are exon terminal domains (3–69 bp, Observed: 76.62%, Expected: 64.26%). Given this enrichment, it is inevitable that exon cores are relatively underrepresented (Observed: 17.89%, Expected: 32.38%; fig. 1*a*). This deviation is highly significant (χ^2^ = 841.64, df = 2, *P* < 1.74 × 10^−^^183^) (supplementary table S3, Supplementary Material online).

To examine whether this biased distribution affects both 5′- and 3′-halves of exons, we perform a similar enrichment analysis considering 5′- and 3′-exon ends separately. The same pattern of significant deviation in pathogenic SNP distribution can still be observed: in both 5′ (χ^2^ = 410.62, df = 2, *P* < 6.85 × 10^−^^90^) and 3′ (χ^2^ = 551.74, df = 2, *P* < 1.55 × 10^−^^120^) half of internal exons, splice sites (≤3 bp) and exon terminal domains (3–69 bp) are preferred regions; and exon cores (>69 bp) are relatively avoided (fig. 1*b*) (supplementary table S3, Supplementary Material online).

We can go further and attempt to estimate the proportion of non-nonsense pathogenic SNPs that have their effects via splicing. If we assume that mutations in exon cores never disrupt splicing, then pathogenic SNP rates in cores provide a background splice-unconnected rate. This assumption is likely to be wrong, not least because splice disrupting mutations in exon cores are described (Woolfe et al. 2010), but renders the method conservative. First, in all disease genes, we estimate the background nonsplicing-related pathogenic SNP rate by asking about the frequency of pathogenic SNPs in exon core regions, defined as the central 100 bp of internal exons longer than 300 bp (there are 707 internal exons longer than 300 bp). The 70,700 (*L*) bp of 100 bp core regions contain 124 (*N*) pathogenic SNPs, which gives us *N*/*L* = 0.001754 SNPs per bp. If 69 bp is considered as exon “end” cutoff, we can then calculate how many pathogenic SNPs we would expect in exon flanks, assuming flanks behave like these cores, this being the core rate per base pair multiplied by the number of exon flank base pairs (2,734,972 bp). This yields an estimate of 4,797 pathogenic SNPs. We actually observe a total of 6,774 SNPs in these exon flank domains (N.B. there are 1,476 pathogenic SNPs in regions outside of these flanks, so 8,250 (6,774 + 1,476) pathogenic SNPs in internal exons). If we assume that the excess at exon ends (6,774 − 4,797 = 1,977 bp) affect splicing, we can calculate the proportion, at 69 bp cutoff exon ends, of all pathogenic SNPs that affect splicing as 1,977/8,250 = 23.97% (supplementary table S4, Supplementary Material online). This method is quite robust to definition of exon end. If we consider different definitions of splice affected exons exon ends in a range from 50 to 100 bp, estimates range from 20% at 50 bp definition of exon end to 27% at 100 bp (supplementary table S4, Supplementary Material online).

The above method is conservative in assuming that splice disrupting mutations only ever occur at exon ends. However, prior estimates (Woolfe et al. 2010) suggest that about 25% of mutations in exon core regions also affect splicing. A more liberal estimate then supposes that the overall background (nonsplice dependent rate) rate is 0.75 × *N*/*L* = 0.001315. This being so, with a total number of base pairs in internal exons of all pathogenic genes (*T* = 3 527 520 bp), we expect *T* × 0.001315 (=4,639) pathogenic SNPs to not exercise their effects via splicing. With a total of 8,250 pathogenic SNPs in all internal exons, the proportion that do exercise their effects via splicing defects we estimate to be (8,250 − 4,639)/8,250 = 43.77%. Both the conservative (∼20%) and liberal estimates (∼43%) support the hypothesis that splice disruption is a major cause of pathology.

Note that we have assumed that nonsense mutations have their effects via the introduction of premature stop codons. However, in principle a stop could also disrupt ESEs and lead to disrupted splicing, possibly leading to the noninclusion of the exon with the mutation. We can then ask about the proportion of all SNPs (including nonsense mutations; *N* = 10,764 SNPs in internal exons; supplementary table S3, Supplementary Material online). Repeating the above calculations for all SNPs distribution in internal exons, we find similar and significant deviated patterns (supplementary fig. S1, Supplementary Material online). These numbers are highly similar to those obtained using just the non-nonsense SNPs. Although nonsense mutations associated with pathology are relatively rare (2,514 SNPs) (supplementary table S3, Supplementary Material online), these too show an excess at exon ends (fig. 1*c*).

### Pathogenic SNPs Are More Likely to Occur in the Terminal Half of Genes

It was previously observed that exons in 5′-positions in genes have higher ESE density than more terminal exons. Comparing second exons to last but one exons within the same gene, for example, it was observed that there is a 2-fold greater ESE density in the former (Wu and Hurst 2015). This it was suggested might reflect the fact that early exons, by definition, have more downstream splice sites than do later ones and hence have a larger number of potential decoy splice sites. Does then 5′- to 3′-position predict pathogenic SNP density? Expectations here are unclear. It might be that 5′-pathogenic SNPs might be more common as they might have a more drastic effect on the subsequent protein. Alternatively, with high ESE density, a given SNP might be less prone to disrupting splicing, the ESEs providing some degree of resilience. A further confounding factor is that if a SNP has a major effect it might be an embryonic lethal and hence not be classified as pathogenic.

In the first instance we divide the genes (not the CDS), into the absolute 5′-first half and the 3′-half (from ATG to stop). We observe that pathogenic SNPs are more likely to occur in the rear (3′) half section of genes (5′-first half: 3,393, 3′-rear half: 6,425; ratio = 6,425/3,393 = 1.89; Binomial Distribution *P* value: 1.74 × 10^−^^209^) (supplementary table S1, Supplementary Material online). However, this analysis neglects the influence of any differential distribution of CDS sequence between first half and terminal half of genes. To address this, we performed a simulation in which we randomly select a pseudo pathogenic SNP (the same nucleotide as the mutating one) in each disease-causing gene. Then, for the whole disease-causing gene data set, we calculate the ratio of number of pseudo SNPs in the 3′-half to that in the 5′-half and take this as the expected ratio to compare with the real observation. After 100 repetitions of the process, we found all simulated ratios (number of 3′-half pseudo SNPs/number of 5′-half pseudo SNPs, mean = 1.7) are less than the observed ratio (ratio = 1.89) (*P* = 0.0099 by 100 randomizations; supplementary table S5, Supplementary Material online).

One might suggest that this need not reflect splicing defects, but rather the lesser impact of mutations in the 3′-half of the CDS. This, however, appears not to be the case. If we consider the distribution in CDS sequences, then there is no significant difference after Bonferroni correction (supplementary table S1, Supplementary Material online). This indicates that relative position within the CDS is not so important, while relative position in the unspliced RNA is. This would be consistent with splice defects being of relevance, but such an interpretation is by no means unique.

### Pathogenic SNPs Preferentially Reside in Proximity to Shorter Flanking Introns

In the human genome, ESE density tends to be higher in the exons flanked by larger introns (Dewey et al. 2006; Cáceres and Hurst 2013; Wu and Hurst 2015). Here, to test if flanking intron size affects distribution of pathogenic SNPs, we consider the relationship between the density of pathogenic SNPs (Dpi = number of pathogenic SNPs/exon length) and flanking intron size.

First, as many exons have no pathogenic SNP, we perform this test only for the exons with pathogenic SNPs. We observe a weak but significant negative correlation between Dpi (supplementary table S6, Supplementary Material online) and the log of flanking intron size (here, “flanking intron” means the “nearest intron”) (Spearman correlation, *ρ* = −0.085, *P* = 1.14 × 10^−^^8^). However, this could be partly due to covariance between exon size and flanking intron size. Indeed, our Spearman’s correlation shows that exon size correlates well and positively with flanking intron size, not only for pathogenic SNP-containing exons (*ρ* = 0.092, *P* = 6.84 × 10^−^^10^), but also for all exons within disease-causing genes (if there is no pathogenic SNP in the exon: Dpi = 0) (*ρ* = 0.037, *P* = 7.94 × 10^−^^10^). Furthermore, as intron sizes are known to covary with gene expression, and gene expression is likely to predict whether a gene is associated with disease (Emilsson et al. 2008), it is also necessary to control for expression level. One way to do this is to perform a covariate controlled analysis, but this depends on the accuracy of the expression data. An alternative method is to perform an intragene analysis, comparing exons within the same gene, which we presume to have the same expression level.

To this end, we set up a simulation in which we randomly select a nucleotide the same as the mutating nucleotide, within the same gene, in internal exons (not including first and last exons) of all disease genes. Then we can ask how many flanking introns (in the same genes as the SNP) of the selected pseudo SNPs are larger than (or equal to) those of real SNPs (binomial test for significance). In every trial of 100 randomization processes, the number of expected flanking introns (flanking intron of pseudo SNPs) that are larger than observed ones (flanking intron of real SNPs) is significantly greater than the number of expected flanking introns that are shorter than observed ones (supplementary table S7, Supplementary Material online, *P* = 0.0099). So, despite covariance with exon size, pathogenic SNPs tend to occur in exons flanked by smaller introns, within the same gene.

To further consider the issue, we considered the density of pathogenic SNPs (Dpi) for first half and terminal half of exons separately (see Materials and Method). We set up a data set of 238 genes that have at least five internal pathologic SNP containing exons. We then perform a Spearman’s correlation analysis between Dpi and flanking (nearest) intron size for each gene. We expect that most intragene comparisons will have a negative correlation (as seen in the between-gene comparison). We find that a significant majority of intragene correlations are indeed negative (Negative correlation: 136, positive correlation: 99; Binomial test *P* = 2.83 × 10^−^^3^; supplementary table S8, Supplementary Material online). We conclude that exonic-disease causing SNPs tend to occur in the vicinity of shorter introns and that this cannot be fully explained by certain possible covariant factors, such as exon size, expression level, and flanking intron selection.

### Pathogenic SNP Distribution Indicates Enrichment Near Zero Phase and 3′ Non-“AGgt” Splice Sites

Usage of tetranucleotide splice sites AGgt and agGT has been found to correlate well and positively with usage of splicing *cis*-motifs across species (Wu and Hurst 2015). Within each of these splice sites, the 2 nt in upper case come from exons. By serendipity we also find that coding phase has a relationship with the usage of tetranucleotide splice sites. This we found through investigating whether there might be a relationship between phase and specific splice site (AGgt and agGT) usage in the human genome. We discovered there to be a significant relation (for AGgt: χ^2^ test: χ^2^ = 308.18, df = 2, *P* = 1.20 × 10^−^^67^, For agGT: χ^2^ test: χ^2^ = 139, df = 2, *P* = 6.57 × 10^−^^31^; supplementary table S9, Supplementary Material online). Through calculating the “fo/fe” value (the ratio of observed number to expected number), we find that AGgt and agGT splice sites tend to be in the phase zero exon ends (supplementary table S9, Supplementary Material online).

We then asked whether splice site phase might also be a predictor of *cis*-motif usage as well as the tetranucleotide splice sites. To test for such a coupling, we performed a phylogenetic correlation analysis employing BayesTraits (Pagel 1999). Across 30 species, we find that exonic splice *cis*-motif usage correlates significantly and negatively (BayesTraits parameter “R Trait 1 2” < 0) with the proportion of phase zero exon ends (table 1). Furthermore, there is also strong evidence for a correlation between proportion of phase zero splice sites and three intronic parameters: *X* (mean CDS length/gene length), *N* (introns per kb exon) and *M* (mean intron size) for each species (table 2; supplementary table S10, Supplementary Material online). The above suggests that there exist trends across species as regards the proportion of a given phase being used and *cis*-motif usage.

**Table 2.** Evidence for Correlation between Proportion of Phase Zero Splice Sites and Splice-Related Genomic Traits.

	*X*	*N*	*M*
**Log BF ****^a^**** (P_p__hase-__zero_ ∼ Splice-related genomic traits)**	58.5625	45.2272	26.6799
**R Trait 1 2****^b^**	>0	<0	<0

Note.—X, mean CDS length/gene length; N, introns per kb exon; M, mean intron size; P_phase-zero_, Proportion of phase zero splice site.

^a^Log BF (log Bayes factor) = 2*(log [harmonic mean (complex model)]-log [harmonic mean (simple model)]), All Log BF values in the table are >10, so the evidences of all correlations are very strong.

^b^BayesTraits parameter “R Trait 1 2” can indicate whether the correlation is positive (>0) or negative (<0).

Since usage of certain tetranucleotide splice sites (AGgt and agGT) and the proportion of zero phase splice site correlates well with ESE usage, do these two predictors of *cis*-motifs usage predict the occurrence of pathogenic SNPs? We address the question using a randomization approach. There are 1,900 SNPs in exons with agGT splice site and 4,534 SNPs flanked by AGgt. To consider the phase of exon ends, there are 1,958 SNPs in exons that are phase 5′ = 0 and phase 3′ = 0 (i.e., symmetric (0,0) exons). We establish the significance of these figures (observed value) by randomly selecting a nucleotide identical to the mutating nucleotide in each specific gene (this can exclude the influence of gene expression level and biased nucleotide content) and, in turn, calculate its exonic end phases and identify the nucleotide content of flanking tetranucleotide splice sites. For all collected pseudo pathogenic SNPs, we obtain the numbers (Expected value) of exons with agGT, AGgt, and phase (5′ = 0, 3′ = 0) separately. We perform this 100 times with *P* value given by *p* = (*n* + 1)/(*m* + 1), where *n* is the number of expected values calculated after randomly selecting pseudo SNPs and meanwhile greater (for phase)/smaller (for AGgt) than (or equal to) the observed value and *m* is 100, this being the number of randomization cycles. We observe that pathogenic SNPs are more likely to be in symmetric (0,0) exons (*P* = 0.0099). The usage of the two specific splice sites seems to have different preferences: for AGgt, observed usage is significantly less than that expected (*P* = 0.0099), meanwhile, agGT has no significant usage pattern (supplementary table S11, Supplementary Material online).

We can also attempt to estimate the degree of enrichment. For all disease-causing genes, we add up the length of all 3′-AGgt splice site internal exons (1,962,796 bp, 55.64%), and calculate the total length of other phase internal exons (1,564,724 bp). The number of SNPs in 3′-AGgt exons is 4,330 (52.48%) and that for other splice site exons is 3,920. This suggests significant avoidance of pathogenic SNPs in 3′-AGgt splice site exons (Observed ratio (52.48%)/ Expected ratio (55.64%) = 0.94) (χ^2^ = 33.33, df = 1, *P* < 7.80 × 10^−^^9^) (supplementary table S12, Supplementary Material online). Similarly, we did enrichment analysis for pathogenic SNPs in symmetric (0,0) internal exons. The length of all such (0,0) exons is 805,767 bp (22.84%) and the total length of other phase internal exons is 2,721,753 bp. As above, the number of SNPs in phase (0,0) exons is 1,958 (23.73%) and that in other phase exons is 6,292. This suggests a weak but significant enrichment of pathogenic SNPs in phase zero exons (Observed ratio (23.73%)/ Expected ratio (22.84%) = 1.04) (χ^2^ = 3.72, df = 1, *P* < 0.05) (supplementary table S12, Supplementary Material online).

Therefore, exons with 3′-splice site being not AGgt and with zero phase at both ends are more likely to contain pathogenic SNPs. As both phase zero exon ends and 3′ non-AGgt splice sites are characteristics of exons with low ESE density, pathogenic SNPs and ESE usage appear to be anti-correlated.

### ESE Density and Pathogenic SNP Density Negatively Covary

Above we have considered several predictors of ESE densities to ask whether in any manner they predict the location of disease-associated SNPs. That disproportionately many pathogenic SNPs occur either at splice sites or at exon ends suggests that disruption of splicing is a key pathogenic process. Similarly, that genes with more exons (controlling for CDS length) are more likely to be disease-associated is consistent with a role for splicing (although this evidence is far from definitive owing to expression level covariance). What is striking, however, is that for our four other predictors, all report that pathogenic SNPs are most common where ESEs are least common: pathogenic SNPs are more common in 3′ gene domains and near short introns, they are associated with splice sites whose phase and nucleotide content are associated with low ESE density.

We might then predict an across-exon correlation between ESE density and density of pathogenic SNPs. To test this prediction we employ a set of ESE motifs with a low false positive rate but a high false negative rate (INT3 set) (Cáceres and Hurst 2013). For disease-associated genes, we calculate ESE density at exonic ends and correlate it with Dpi of these exons (see Materials and Method). A weak but significant negative correlation is found using Spearman’s correlation (*ρ* = −0.016, *P* = 0.009) and Goodman–Kruskall gamma test (*γ* = −0.023, *P* = 0.004) (supplementary table S13, Supplementary Material online), the latter being less sensitive to multiple tied entries. This is consistent with the notion that pathogenic SNPs tend to be at ends of exons with low ESE density.

## Discussion

One rationale for our findings is that many non-nonsense pathogenic SNPs disrupt splicing and that splice disruption is 1) more likely for mutations at exon ends and 2) more likely when a given exon has relatively few ESEs to provide robustness to the loss of any one. Thus, exon ends are hot spots of disease-associated mutations as these are where splice disrupting mutations occur. Likewise, genes with many potential alternative splice sites (those with many exons controlling for CDS length) are those more prone to be disease-associated. Within genes different parts are more ESE reinforced than others and an exon with a high density of ESEs might, for example, have multiple alternative ESE motifs to attract any given SR protein, should any given ESE mutagenically “fail.” Conversely, some exons, notably those with low ESE density, are more prone to fail to splice correctly and these exons appear to be the hotspots for pathogenic SNPs. This we call the fragile exon model. Such a notion has precedent. For example, it has been observed that exons lacking a downstream intronic poly-G run, a known intronic splice enhancer, are more sensitive to 5′-splice site mutations (Lu et al. 2011). More generally, it will be informative to ask whether intronic SNPs that disrupt splicing (Kawase et al. 2007; Perriaud et al. 2014) are more likely to occur in proximity to low ESE density fragile exons.

The fragile exon model, however, need not be the only framework within which to interpret our data. As we are here analyzing pathogenic SNPs we must be alive to the notion that the population of SNPs is not random. It is possible, for example, that exons with more ESEs are as likely to be disrupted by SNPs (i.e., as fragile) but when such disruption occurs the effects are so dramatic (e.g., early embryonic lethality) that the SNP is never called “pathogenic.” Indeed, were there exons whose correct splicing is so crucial, we might expect them to be supported by a very high density of ESEs to enable correct splicing in the absence of mutations. To differentiate between such models, one would need experimental evidence looking at the rate of missplicing in ESE-rich and ESE-poor exons within the same gene. If the rate is the same in the two then the second model might be more parsimonious. If the rate of missplicing is higher in the low ESE class then the “fragile” exon model is more parsimonious.

We predict that 20–45% of SNPs are pathogenic owing to their effects on splicing. Interestingly, we see a similar trend for nonsense SNPs also to be enriched at exon ends (fig. 1*c*). This suggests that either nonsense mutations may exert their effects by mechanisms beyond introduction of a premature stop codon or that an aspect of our methodology to derive the above estimates may be incorrect. We assume that the excess of pathogenic SNPs at exon ends can be explained by the greater sensitivity of such domains to splice-altering mutations, as previously demonstrated (Woolfe et al. 2010). The extent of the excess then depends on what one considers the background rate. While our assumption appears well defended, we can nonetheless ask whether there might be other causes.

The process of nonsense-mediated decay (NMD) might also affect the rate of nonsense mutations that cause disease as a function of proximity to exon–exon junctions. This is because there is a gap of about 55 bp downstream of a premature stop codon that will not trigger NMD if there is an intron is located within this gap (Nagy and Maquat 1998). However, the end of exon enrichment for pathogenic SNPs is seen for non-nonsense SNPs as well as for nonsense ones, so rendering this an ungeneral explanation. A further possibility is that there might be a higher de novo mutation rate at exon ends for reasons unknown (association with nucleosomes might be conjectured [Kogan and Trifonov 2005; Chen et al. 2012]). This, however, appears unparsimonious as SNP rates at 4-fold degenerate sites not belonging to putative ESEs are if anything slightly lower at exon ends than cores (Cáceres and Hurst 2013). The fact that the rate appears very slightly lower might reflect a lower mutation rate (contra to what is required to explain the excess of end of exon pathogenic SNPs) or possibly reflects the imprecise definition of what constitutes an ESE. Where some true ESE hexamers left in the “non-ESE” class then they should be subject to the same purifying selection as witnessed for the true ESE hexamers. Given this uncertainty, direct parent-offspring sequencing to infer mutation rates as a function of intragene position would be a worthwhile enterprise.

## Materials and Methods

### Derivation of Exon and Intron Sequences from 30 Species

From “Table Browser” of UCSC (Karolchik et al. 2004) (http://genome.ucsc.edu/cgi-bin/hgTables, last accessed January 23, 2014) and FTP site of NCBI (ftp://ftp.ncbi.nlm.nih.gov/genomes, last accessed January 23, 2014), we obtained all available genes from 30 species (*Anolis carolinensis, Anopheles gambiae, Arabidopsis thaliana, Brachypodium distachyon, Caenorhabditis elegans, Callithrix jacchus, Cryptococcus neoformans, Dictyostelium discoideum, Drosophila melanogaster, Danio rerio, Ectocarpus siliculosus, Gallus gallus, Gorilla gorilla, Homo sapiens, Ictidomys tridecemlineatus, Meleagris gallopavo, Macaca mulatta, Mus musculus, Oryzias latipes, Oryza sativa, Pongo abelii, Plasmodium falciparum, Paramecium tetraurelia, Pan troglodytes, Saccharomyces cerevisiae, Schizosaccharomyces pombe, Strongylocentrotus purpuratus, Sus scrofa, Takifugu rubripes, and Xenopus tropicalis*). Sequences without normal start codon (ATG) and stop codons (TAA, TAG, and TGA), the genes with internal stop codons, ambiguous nucleotides (“N”), and those without introns were all removed from the data set.

### Collection of Information about Pathogenic SNPs

We downloaded disease-related information of sequence variation from Clinvar database (http://www.ncbi.nlm.nih.gov/clinvar/, last accessed November 24, 2014) (Landrum et al. 2014). In total, 9,818 pathogenic missense and silent mutations SNPs (which together we consider as non nonsense SNPs) were selected for further investigation (supplementary table S1, Supplementary Material online). From UCSC (http://genome.ucsc.edu/cgi-bin/hgTables, last accessed November 24, 2014), 1,747 gene sequences that contain pathogenic SNPs were derived (supplementary table S2, Supplementary Material online).

### Comparison of Quantity of Exons between Disease Genes and Nondisease Genes

We compare the number of exons in disease-associated genes (1,747 genes) and nondisease genes (38,474 genes). To control for the effect of CDS length, we analyzed the residuals from loess regression of number of exons predicted by CDS size (supplementary table S2, Supplementary Material online). By Mann–Whitney *U* test analysis on the residuals of these two kinds of genes, we can assay whether number of exons in disease-associated genes is significant different from that of nondisease genes when CDS is controlled (supplementary table S2, Supplementary Material online).

### Investigation of the Distribution of Pathogenic SNPs

By using our custom programs, we observed the distribution of pathogenic SNPs in exons or genes. The relevant information about these exons were also obtained, such as phase of exon ends, splice sites, distance from SNPs to the nearest splice sites, and flanking intron size (the flanking intron here is the nearest one to the SNP, if SNP is in the middle position, the flanking intron refers to the longer one; supplementary table S1, Supplementary Material online).

To test if pathogenic SNPs tend to be at end of exons, we observed their distribution in internal exons. Three domains (“≤3 bp,” “3–69 bp,” and “>69 bp”) were considered for enrichment of pathogenic SNPs. To establish the significance of the distributions, we summed up, by each SNP bearing exon, the length of each domain and divided them by total length of all the pathogenic exons separately thereby deriving the expected distribution (fig. 1; supplementary table S3, Supplementary Material online). We performed a similar enrichment analysis in order to exclude the effect coming from 5′ to 3′ distribution bias. We investigated the distribution of pathogenic missense and silent SNPs in 5′- and 3′-exon ends separately. A chi-squared test was used to examine the significance of all these results with fo/fe being employed to detect whether the domain is preferred (>1) or disliked (<1) (supplementary table S3, Supplementary Material online).

### Estimation of the Proportion of Splice-Affecting Pathogenic SNPs at Exon Ends by the Background Removal Method

Assuming that mutations in exon cores never disrupt splicing, we defined the central 100 bp sequences (total length is “L”) of internal exons longer than 300 bp as core regions and calculated the background nonsplicing related pathogenic SNP (total number is “*N*”) rate by proportion (*N*/*L*) of pathogenic SNPs in this core region.

First, we consider 69 bp as exon end cutoff and calculate the total length of end regions in all internal exons (*M*). Then the predicted number of nonsplicing related SNPs is *P* = *M* × *N*/*L*. After obtaining the number of all pathogenic SNPs at exon ends (Q), we can estimate the proportion of pathogenic SNPs that affect splicing at exon ends by *P*s = (*Q* − *P*)/(*N* + *Q*) (supplementary table S4, Supplementary Material online). If we consider that splice disrupting mutation in core occurs at an approximately 25% rate (Woolfe et al. 2010), the background nonsplicing related pathogenic SNP rate is 0.75 × *N*/*L* (supplementary table S4, Supplementary Material online).

To examine whether the definition of exon end length affects robustness of this background removal method, we changed exon end cutoff from 50 to 100 bp by 5 bp every step and observed the change of proportion of splice-affecting pathogenic SNPs at exon ends (supplementary table S4, Supplementary Material online).

### Biased Location of Pathogenic SNPs in 5′- and 3′-Halves of Genes

We observed the distribution of pathogenic SNPs in the absolute 5′-first half and the 3′-half (from ATG to stop) of each gene (supplementary table S1, Supplementary Material online). To consider the influence from the biased distribution of CDS sequence between first half and terminal half of genes, we performed a randomization. First, we randomly selected pseudo pathogenic SNPs in each disease-causing gene according to the real mutating nucleotides. Second, by calculating the ratio (expected values) of the number of pseudo SNPs at 3′-half to that at the 5′-half, we compared this expected ratio with that observed (ratio = 1.89). After repeating this comparison 100 times, we examine significance of this randomization process by *P* = (*n* + 1)/(*m* + 1), where *n* is the number of expected values calculated after randomly selecting pseudo SNPs and meanwhile greater than (or equal to) the observed value and *m* is the number of randomization cycles 100 (supplementary table S5, Supplementary Material online). To consider the impact of mutations in the 3′-half of the CDS, we did analysis of the distribution of disease-causing SNPs in CDS sequence, testing for significance after Bonferroni correction (supplementary table S1, Supplementary Material online).

### The Relationship between the Distribution of Pathogenic SNPs and Flanking Intron Size

An index of pathogenic SNP density (Dpi = number of pathogenic SNPs/exon length) was introduced to measure how easily a specific exon causes disease by pathogenic SNPs (supplementary table S6, Supplementary Material online). We performed Spearman’s correlation analysis between Dpi and the log of flanking intron size only for disease SNP bearing internal exons. Here, flanking intron means the nearest intron. If the SNP happened to be in the middle then flanking intron is the longer one of the two equidistant introns.

Furthermore, to control for exon size and gene expression covariance we set up a simulation by randomly selecting pseudo SNPs according to the mutating nucleotide in internal exons (not including first and last exons) with the same gene. Then we determined the flanking intron size of the selected pseudo SNPs. By comparing the pseudo flanking intron size with the real one, we can determine how many randomly selected flanking introns are larger or smaller than the flanking introns of real pathogenic SNPs (result of each randomization simulation gives significance by Binomial Test). Repeating this trial 100 times allows estimation of *P* value from p = (*n* + 1)/(*m* + 1), where *n* is the number of trials in which count of larger pseudo flanking intron size is greater than count of smaller ones and *m* is 100 (supplementary table S7, Supplementary Material online).

Additionally, because one exon is flanked by two introns on both sides, here, to consider Dpi value without any effect from flanking intron selection, we calculated Dpi values for the first half and the terminal half separately (5′-Dpi = number of pathogenic SNPs in 5′-half of exon/half length of exon, 3′-Dpi = number of pathogenic SNPs in 3′-half of exon/half length of exon). Genes (*N* = 238) that have at least five different internal exons containing pathogenic SNPs were selected to perform Spearman’s correlation analysis between Dpi and flanking intron size. Based on these Dpi and flanking intron size of the 238 genes, we calculated ρ values from correlation analysis for each gene. Moreover, by Binomial distribution test, we can test if negative correlations tend to more common than expected by chance (supplementary table S8, Supplementary Material online).

### Correlation between *cis*-Motif Usage with the Proportion of Phase Zero Splice Sites

For human genes, we calculated the number of splice sites in different phases (0, 1, 2) and the sum of numbers of all splice sites as a function of phase. A chi-squared test was used to examine if there is relation between coding phases and splice site usage (supplementary table S9, Supplementary Material online).

For comparative analysis we investigated all splice sites of 30 species, and classified them as “phase zero splice sites” (i.e., splice site in exons end with coding phase = 0) and “phase nonzero splice sites” (coding phase = 1 or phase = 2) (supplementary table S10, Supplementary Material online). Given that the composition of experimentally defined ESEs predicts the codons preferred near exon ends (Parmley and Hurst 2007; Cáceres and Hurst 2013), we presume that the frequency of distorted codon or amino acid usage in vicinity of exon junctions is a fair measure of *cis-*splice motif usage (Wu and Hurst 2015). To accord with an earlier analysis (Warnecke et al. 2008), the trend in usage of each codon and amino acid was investigated as a function of the distance from the exon–intron boundary up to a distance of 34 codons. The 5′- and 3′-ends were analyzed separately with the codon in direct proximity to the boundary being eliminated and the first and last exons being excluded. For each codon and amino acid under consideration, we determined, after Bonferonni correction, *ρ* and *P* value by two-tailed Spearman’s correlation of proportional usage as a function of distance from the boundary. For each species we then calculated the proportion of codons or amino acids showing significant skew both at 5′- and 3′-ends across all exons and consider this the metric of *cis*-motif usage for that species. We calculated this metric by sampling 5,000 exons, so that there is no effect of the number of exons in different species, and a sample size uncorrected method, as we did in previous paper (Wu and Hurst 2015).

Based on the data set of 30 species genes, we also calculated the intronic parameter X (mean CDS length/gene length), which is an aggregate measure of intron size and density, for each species (supplementary table S10, Supplementary Material online). To allow for phylogenic nonindependence between data points, the program “Continuous” of BayesTraits (Pagel 1999) was used to study correlations between *cis*-motif usage and proportion of phase zero splice sites (supplementary table S10, Supplementary Material online) by a Markov chain Monte Carlo method. The phylogenetic tree with branch lengths is as previously employed (Wu and Hurst 2015). We abstracted the last harmonic mean from the result file, and took it as an estimation of marginal likelihood, to calculate the “Log BF” value and further test whether there is evidence for the correlation after phylogenetic correction (table 1).

To confirm the relationship between *cis*-motif usage and the proportion of phase zero splice site, we also correlated the proportion of phase zero splice site with three parameters: *X* (mean CDS length/gene length), *N* (introns per kb exon) and *M* (mean intron size) across species (table 2; supplementary table S10, Supplementary Material online).

#### Preference of Pathogenic SNPs for Splice Sites and Exon End Coding Phase

We explored the phase of exon ends and the tetranucleotide splice sites of the internal exons with pathogenic SNPs. Through randomly selecting a pseudo SNP (identical nucleotide with the mutating nucleotide) in each gene and scanning the exonic end phase and tetranucleotide splice sites of this pseudo SNP located exon, we test the significance of the real figures (observed value).

For all collected pseudo pathogenic SNPs, the numbers (Expected value) of exons with agGT, AGgt, and symmetric (0,0) exons were calculated separately. We repeated the randomization 100 times, and *P* value was obtained by the formula *p* = (*n* + 1)/(*m* + 1), where *n* is the number of expected values calculated after randomly selecting pseudo SNPs and that are less than (or equal to) the observed value and *m* is 100 (the number of times of repeatedly performed) (supplementary table S11, Supplementary Material online).

We also do an assessment of the relative enrichment of pathogenic SNPs in (0,0) internal exons and 3**′-**AGgt splice site internal exons. The expected ratio is the length of all (0,0) (or 3**′-**AGgt splice site) internal exons divided by length of total internal exons. Similarly, the observed ratio is the number of pathogenic SNPs in (0,0) (or 3**′-**AGgt splice site) exons divided by total number of pathogenic SNPs. The metric of enrichment is “Observed ratio/Expected ratio” with significance being tested by a chi-squared Test (supplementary table S12, Supplementary Material online).

### Correlation between Dpi and Exon End ESE Density in Disease-Associated Genes

To calculate ESE density, we employ an ESE candidate data set (INT3) composed of 84 *6-mer* (hexamer) motifs. This data set is likely to have a low false positive rate but a high false negative rate because it is an intersect of at least three well-identified ESE data sets (Cáceres and Hurst 2013). The list of INT3 hexamers may be obtained from supplementary table 1, Supplementary Material online, of the above article at http://www.genomebiology.com/content/supplementary/gb-2013-14-12-r143-s1.xlsx. Based on this INT3 data set, we calculate ESE densities of each internal exon end and, for exons longer than 138 (69 × 2) bp, take mean ESE density value of two 69 bp ends at both sides of exon (if exon is shorter than 138 bp, then the whole exon are regarded as end region) for correlation analysis with Dpi. Significance of the correlation is examined by Spearman’s correlation and Goodman–Kruskall gamma test (supplementary table S13, Supplementary Material online, if there is no pathogenic SNP in a exon, Dpi of this exon = 0).

## Supplementary Material

Supplementary tables S1–S13 and figure S1 are available at *Molecular Biology and Evolution* online (http://www.mbe.oxfordjournals.org/).

Supplementary Data
