# Supplementary material for: Determinants of the Usage of Splice-Associated cis-Motifs Predict the Distribution of Human Pathogenic SNPs
Source: Mol Biol Evol. 2015 Nov 5;33(2):518–29. doi: 10.1093/molbev/msv251 (PMC4866546; doi:10.1093/molbev/msv251)
Supplement: Supplementary Data [file supp_33_2_518__index.html]

Supplementary Data 

# Determinants of the Usage of Splice-Associated *cis*-Motifs Predict the Distribution of Human Pathogenic SNPs

## Supplementary Data

files

- Supplementary Data - xlsm file
- Supplementary Data - pdf file
