# Supplementary material for: Determinants of the Usage of Splice-Associated cis-Motifs Predict the Distribution of Human Pathogenic SNPs
Source: Mol Biol Evol. 2015 Nov 5;33(2):518–29. doi: 10.1093/molbev/msv251 (PMC4866546; doi:10.1093/molbev/msv251)

Supplementary Fig. S1.

Distribution of all pathogenic SNPs (nonsense, missense and silent mutations) in internal exons

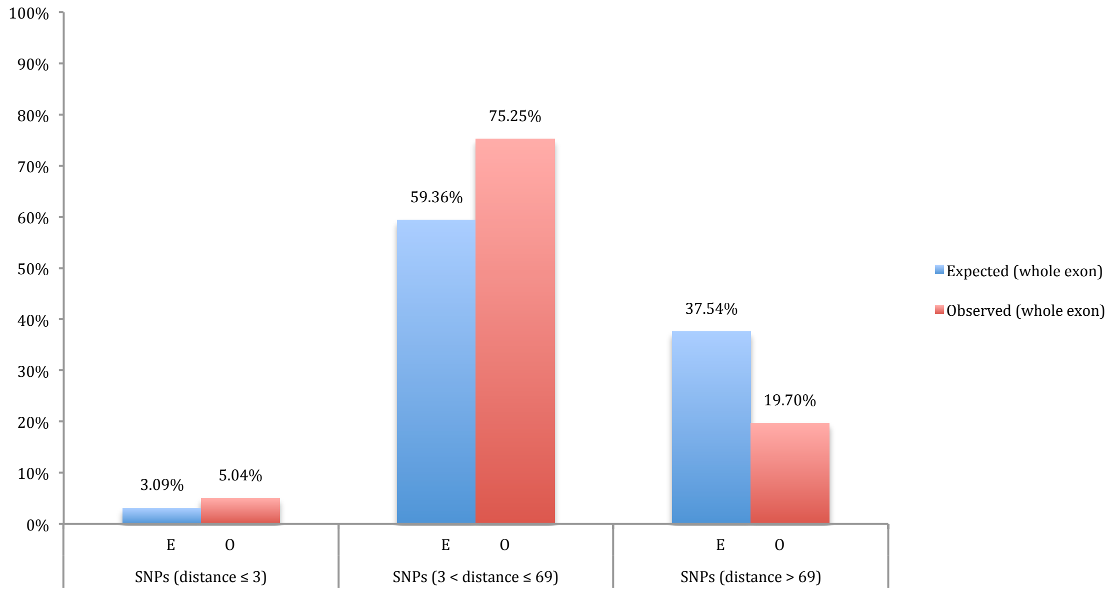

Supplement: Supplementary Data [file supp_msv251_supplementary_fig_S1.pdf]
